# Invasion and effective size of graph-structured populations

**DOI:** 10.1371/journal.pcbi.1006559

**Published:** 2018-11-12

**Authors:** Stefano Giaimo, Jordi Arranz, Arne Traulsen

**Affiliations:** Evolutionary Theory Department, Max Planck Institute for Evolutionary Biology, Plön, Germany; UC Davis, UNITED STATES

## Abstract

Population structure can strongly affect evolutionary dynamics. The most general way to describe population structures are graphs. An important observable on evolutionary graphs is the probability that a novel mutation spreads through the entire population. But what drives this spread of a mutation towards fixation? Here, we propose a novel way to understand the forces driving fixation by borrowing techniques from evolutionary demography to quantify the invasion fitness and the effective population size for different graphs. Our method is very general and even applies to weighted graphs with node dependent fitness. However, we focus on analytical results for undirected graphs with node independent fitness. The method will allow to conceptually integrate evolutionary graph theory with theoretical genetics of structured populations.

## Author summary

Evolving populations, companies, social circles are networks in which genes, resources and ideas circulate. Imagine a useful novelty is introduced in the network: a favorable mutation, a new product concept or a smart idea. Will this novelty be retained and propagated through the network or rather lost? Using tools originally devised for demographic research, we model the dynamics of the very initial spread of a useful novelty in a network. The network structure has a strong impact on these dynamics by affecting the effective network size through random effects. This effective network size, which correlates with the probability that a novelty spreads and is different from the actual size (i.e. number of nodes), varies with network structure. The effective size can even become independent of the actual network size and thus remain very small even for huge networks.

## Introduction

Evolutionary graph theory studies populations on graphs [1]. It aims at capturing an evolving population with explicit spatial connections, e.g. individuals occupying neighboring patches. Moreover, it is frequently argued that it also applies to cultural evolution in a company or a social network. The main quantity of interest for graphs has been the probability of fixation of a mutation [1–5] and the associated time [6–8]. Often graph structures are classified as amplifiers or suppressors of selection, depending on whether a beneficial mutation is more or less likely to get fixed in comparison to a complete graph [1, 5, 9]. The underlying forces driving fixation have been subject to less investigation [4, 10]. Yet, in order to develop a more general understanding of the process of fixation, it would be relevant to study what are the graph properties that contribute to modulate the strength of selection.

Here, we look at graphs via the matrix population modeling approach, which was developed for demographically structured populations [11]. In these populations, individuals are classified according to the stage of life (e.g. age, size) they are in. Attributing to each graph node the formal role that in matrix population models is given to a life stage, it is possible to define matrix population models for evolutionary graphs. This structural analogy expands upon a connection between evolutionary graph theory and evolutionary demography that was already suggested in [12] and, retrospectively, in [13]. The present work explores and deepens this connection to study the dynamics of invasion and drift in graphs relating graph quantities with familiar quantities from the genetics of structured populations. In the Methods section, we describe the evolutionary process on graphs in a general way. In doing so, we introduce a matrix notation that helps to keep track of the expected change in population composition in the neutral graph, i.e. when it hosts a single type of individuals. In the Results section, we show that this matrix can be given the interpretation of a matrix population model for a graph. Subsequently, we extract reproductive values on graphs from this model. We propose how to define a scalar measure of fitness and how to build a matrix model that captures mutant invasion on graphs. Finally, we suggest how invasion analysis along with other quantities of demographic nature can be used to obtain an educated guess of mutant fixation probabilities on graphs.

## Methods

An evolutionary graph is a weighted directed graph where each node hosts one individual. The graph structure is constant, has no loops and is strongly connected, which means that any node can be reached from any other node. Individuals can be either of resident type or of mutant type and no individual can change of type. Individual fitness depends on type and node. The population on the graph gets updated in discrete time. Each update event keeps the size of the population constant and comprises a selection step, i.e. one individual is chosen, and a reproduction step, i.e. one individual replaces one neighbor with an offspring. Offspring are of the same type as their parent and replaced individuals are removed from the graph. We consider two update processes (see S1 Text): Birth-death (Bd) and death-Birth (dB), where the capital letter signals where fitness matters. Under Bd, first a single individual is chosen with probability proportional to its fitness. The chosen individual then replaces one neighbor with an offspring with probability proportional to the weight of the connecting link. Under dB, a random individual is removed first. Then, a neighbor is chosen to place an offspring in the vacant node with probability proportional to the product between fitness and weight of the connecting arrow. We define

$$\begin{array}{c} y^{i,j}=\text{Pr}\left(i\;\text{dies}\;\text{and}\;\text{is}\;\text{replaced}\;\text{by}\;\text{the}\;\text{offspring}\;\text{of}\;j\right) \end{array}$$ (1)

As the graph has no loops, no one can replace itself and, therefore, we always have *y*^*i*, *i*^ = 0. It is useful to define the set *M* ⊆ {1, 2, …, *N*} giving the current configuration of mutants in the graph. For example, *M* = {3, 10} means that there are two mutants, one in node 3 and one in node 10. In this way, we can write explicitly *y*^*i*, *j*^(*M*) to indicate that the probability to be replaced depends on population composition.

To track the change of the population composition over one time step we represent the event occurring with probability *y*^*i*, *j*^ with the matrix **A**^*i*, *j*^. This matrix is identical to the identity matrix, except for two entries in row *i*: (i) The entry in (*i*, *i*) is zero, which indicates the death of the individual in node *i*; (ii) The entry at (*i*, *j*) is one, which means that the individual in *i* at *t* + 1 is contributed by the individual in *j* at *t*. Each 1 on column *j* along the diagonal denotes survival from *t* to *t* + 1 of the individual in node *j*. A zero in entry (*k*, *m*) indicates that the individual in node *k* at *t* + 1 is not contributed by the individual in node *m* at *t*. For our purposes, we form the average matrix $A=\left[a_{i,j}\right]=\sum_{i,j}\left(y^{i,j}\left(⌀\right)A^{i,j}\right)$, where ⌀ is the empty set. This matrix reports expected dynamics over one time step in the absence of mutants. Looking at **A** entrywise,

$$\begin{array}{c} a_{i,j}=\{\begin{array}{cc} y^{i,j}\left(⌀\right) & i\neq j,\\ 1-\sum_{k}y^{i,k}\left(⌀\right) & i=j. \end{array} \end{array}$$ (2)

**Fig 1:**
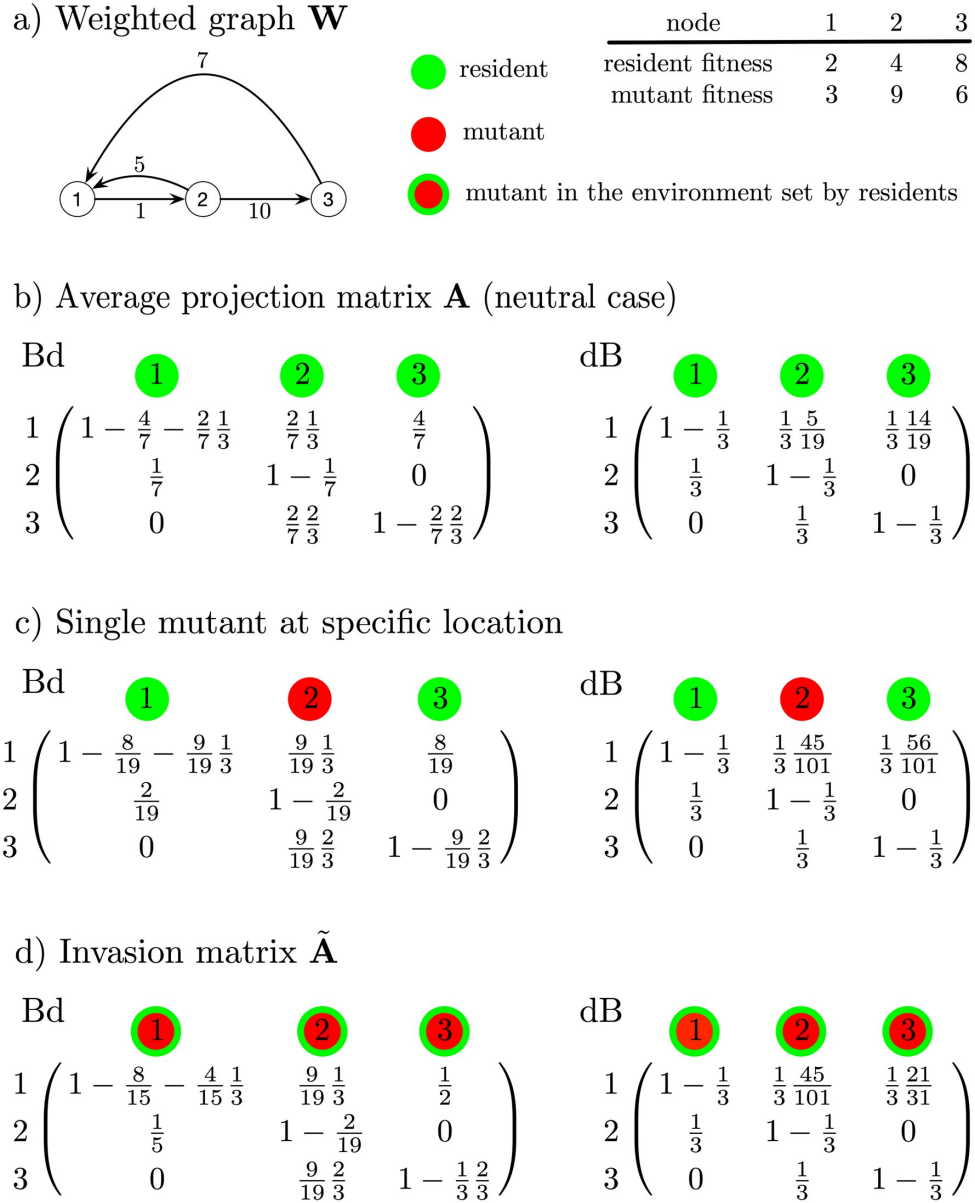
Example of matrix population models for graphs. (a) The population resides on the graph **W**. (b)-(d) We build all relevant matrix population models for both the Bd process (left) and the dB process (right). Each matrix column represents a node. (b)-(c) A column reports the per-time-step probability that the corresponding node contributes the individual in the row node. (b) Matrices correspond to the average matrix model in the neutral case (i.e. only residents). (c) Matrices correspond to the expected matrix model in the presence of a single mutant at node 2. (d) Matrices correspond to invasion matrices. For invasion, the column reports the per-time-step probability that the corresponding (mutant) node contributes the individual in the row node assuming all nodes other than the focal column node are resident. Comparing (c) and (d) helps understanding how invasion matrices are constructed: column 2 for each update process is shared by the matrices. The invasion matrix is built by binding the mutant columns obtained from the construction of a matrix like the one in (c) for each possible mutant position.

Note that *a*_*i*,*i*_ is the probability that no neighbor of *i* reproduces into *i*. Under dB update, $a_{i,i}=1-\frac{1}{N}$, i.e. the probability that *i* is not chosen to die. The matrix **A** is row stochastic. Intuitively, row *i* is a distribution assigning to each (column) node the probability to be the one contributing the individual in *i* in the next time step. As the graph is strongly connected by assumption, **A** is also irreducible [14] and has Perron root λ = 1. An example of construction of **A** for both update processes is in Fig 1.

## Results

### Reproductive value on graphs as an eigenvector

The matrix **A** captures the expected change in the neutral graph, i.e. when there is a single type of individuals. This matrix is structurally analogous to matrix population models that serve to capture the dynamics of demographically structured populations. Organisms typically have a life cycle. This is the collection of stages (e.g. age, size) the organism can go through together with a specification of the stage-specific capacity of generating offspring and of the probabilities of transitioning through stages. A so-called matrix population model represents a life cycle and can be used to model a population of the organism of interest [11]. A vector of current stage abundances **n**(*t*) can be left multiplied by the current matrix model **B**(*t*) = [*b*_*i*,*j*_(*t*)], where entry *b*_*i*,*j*_(*t*) is the number of individuals in *i* at *t* + 1 per individual in *j* at *t*, so that *b*_*i*,*j*_(*t*)≥0 and the vector **n**(*t* + 1) = **B**(*t*)**n**(*t*) is obtained. For large populations with constant life cycle, **B** is constant. If **B** is also irreducible, it has a Perron root λ [11] and, asymptotically, the vector **n** becomes proportional to the right eigenvector **u**_**B**_ of **B** corresponding to λ. The population thus acquires a stable stage distribution, called demographic equilibrium. At this equilibrium, the population increases exponentially in size when λ > 1, it is stationary when λ = 1, and it decreases in size when λ < 1. For graphs, entry (*i*,*j*) of **A** gives the expected number of individuals in *i* at *t* + 1 per individual in *j* at *t*. The matrix **A** has Perron root of 1, which is consistent with the fact that the population size on the graph is of constant size. Demographically, a graph is always at demographic equilibrium: as for any row stochastic matrix, a right eigenvector of **A** corresponding to λ is $u=\frac{1}{N}{\left[1,1,...,1\right]}^{T}$, where the superscript *T* indicates vector transposition. Therefore, we suggest that **A** can be interpreted as a matrix population model.

The first consequence of this interpretation is the possibility of straightforwardly obtaining reproductive values for graphs. In general, the left eigenvector $v_{B}^{T}$ corresponding to the Perron root λ of a matrix population model **B** is the reproductive value vector [11, 15]. The *j* component of $v_{B}^{T}$ measures the relative contribution of stage *j* individuals to the ancestry of the future population [11, 15]. The reproductive value at *j* can also be thought of as the residual expected number of offspring to an organism currently in stage *j* where offspring are weighted by their stage of birth and cumulated population growth [15]. For the scaling of the eigenvectors, we assume in the following

$$\begin{array}{c} \begin{array}{c} \sum_{i}u_{i}=1,\\ \sum_{i}u_{i}v_{i}=1. \end{array} \end{array}$$ (3)

In the case of a graph, the left eigenvector **v**^*T*^ of **A** generalizes the notion of reproductive value as defined for undirected graphs by [12] to any graph. This generalization corresponds to that first given in [16]. As in the demographic case, the component *j* of **v**^*T*^ gives the expected contribution of node *j* to the ancestry of the future population. It was already noted in [12, 16] that the reproductive value of a node is proportional to the fixation probability of a neutral mutation initially introduced at that node as a single copy in the graph. This is a special case of a known result in the evolutionary demographic framework [13, 17, 18].

### Neutral fitness

As fitness may be node dependent, a comparison of mutant and resident fitness vectors may not be easy. Hence, we suggest a scalar measure of fitness using the demographic notion of reproductive value that was introduced in the previous section. In particular, we use reproductive values to weight individuals in the graph. This weighting is needed in any structured population to account for the fact that individuals in different stages may have different future prospects of surviving and reproducing and they cannot be counted as all equal [15, 19, 20]. The rationale for this is given by the asymptotic meaning of reproductive value as a quantity proportional to neutral fixation probability, as previously explained. By our scaling (3) and the fact that $u_{i}=\frac{1}{N}$, the total reproductive value in the graph always equals the population size, i.e. ∑_*i*_ *v*_*i*_ = *N*. We then define neutral RV-fitness as the expected contribution to the total reproductive value in one time step of a random individual in a neutral population. The expected contribution of an individual in node *j* to reproductive value in the next time step is the expected number of individuals contributed to the next time step (either by surviving or by producing offspring), where each contributed individual is weighted by its reproductive value. The matrix population model tells us that there are *a*_*i*,*j*_ expected individuals in node *i* in the next step per individual in node *j*, and each of these expected individuals has reproductive value *v*_*i*_. As reproductive value is a left eigenvector (with corresponding eigenvalue λ = 1 in graphs), the expected contribution of an individual in node *j* to reproductive value in the next time step is precisely reproductive value at *j*, i.e. *v*_*j*_ = λ*v*_*j*_ = ∑_*i*_ *v*_*i*_ *a*_*i*,*j*_. Neutral RV-fitness *v* is the average of this expectation in the population. By our normalization (3), neutral fitness is then 1.

### Invasion

In infinite populations, invasion analysis is a tool to study the initial fate of a rare mutant. The focus is on mutant fitness in the environment set by residents so that mutant frequency is close to zero [21]. If this invasion fitness is greater than the neutral fitness, then the mutant can reach a higher than vanishing frequency. It is not straightforward to perform a similar analysis for finite populations [22], where mutant frequency is always at least $\frac{1}{N}$. Typically, this situation has been dealt with by developing new and more complex measures of evolutionary stability [23, 24]. Here, we instead propose a method to perform invasion analysis on graphs that is rooted in evolutionary demography.

We propose to look at whether a single mutant that may appear in any one node has higher than neutral RV-fitness. Suppose the mutant is very similar to the resident, i.e. weak selection. If the mutant is in *j*, its expected contribution to reproductive value at *t* + 1 is ∑_*i*_ *v*_*i*_(*a*_*i*,*j*_ + Δ*a*_*i*,*j*_), where Δ*a*_*i*,*j*_ are mutant deviations from resident values and reproductive values are still those of the neutral population. Mutant deviations are computed accounting for the fact that *j* is the only mutant. Averaging over all nodes, the expected contribution to reproductive value $\tilde{v}$ of a random mutant is

$$\begin{array}{c} \begin{array}{cc} \tilde{v} & =\frac{1}{N}\sum_{i,j}v_{i}\left(a_{i,j}+\Delta a_{i,j}\right)\\ & =\sum_{i,j}v_{i}\left(a_{i,j}+\Delta a_{i,j}\right)u_{j}\\ & =v^{T}Au+v^{T}\Delta Au\\ & =1+\sum_{i,j}\frac{\partial \lambda }{\partial a_{i,j}}\Delta a_{i,j}. \end{array} \end{array}$$ (4)

In the second line we use the fact that $\frac{1}{N}$ corresponds to the neutral stable distribution of individuals on nodes *u*_*j*_ for all *j*, in the third line we let Δ**A** be the matrix containing all mutant deviations, and in the last line we use the relationship [11],

$$\begin{array}{c} \frac{\partial \lambda }{\partial a_{i,j}}=v_{i}u_{j}. \end{array}$$ (5)

The intuition behind (5) is that the (*i*,*j*) entry of the matrix population model regulates the flux of individuals that enter stage *i* and are contributed by individuals in stage *j*. When the value of such entry is modified, the effect on λ, which demographically represents stable population growth, is influenced by how many individuals are in stage *j*, which is captured by the *u*_*j*_ (i.e. stable fraction in *j*) factor, and by how much individuals in *i* influence population dynamics, which is captured by the *v*_*i*_ (i.e. reproductive value of *i* individuals) factor. Hence, we take mutant invasion to be successful whenever $\tilde{v}>v=1$, i.e. at the beginning of the invasion a deviating mutant has a higher expected contribution to reproductive value in one time step than a neutral mutant.

We can also define the matrix $\tilde{A}=A+\Delta A$, where each column *j* contains the survival and reproductive abilities of a single mutant in *j* when everyone else is a resident. Looking entrywise at this matrix,

$$\begin{array}{c} {\tilde{a}}_{i,j}=\{\begin{array}{cc} y^{i,j}\left(\left\{j\right\}\right) & i\neq j,\\ 1-\sum_{k}y^{i,k}\left(\left\{i\right\}\right) & i=j. \end{array} \end{array}$$ (6)

Note that, under dB, we have ${\tilde{a}}_{i,i}=1-\frac{1}{N}$, as death is always uniformly random. An example of construction $\tilde{A}$ under both update processes for a weighted graph with 3 nodes is in Fig 1.

The matrix $\tilde{A}$ is, like **A**, nonnegative and irreducible and has Perron root $\tilde{\lambda }$, which thus corresponds to the spectral radius of this matrix. The argument in (4) shows that $\tilde{v}=\tilde{\lambda }$. Therefore, we can define the selective RV-advantage to be $\Delta \lambda =\tilde{\lambda }-1$. As *v* = λ = 1, this tells us whether mutant invasion will be successful (Δλ > 0), neutral (Δλ = 0) or unsuccessful (Δλ < 0). Finally, it may be noted that the proposed form of invasion analysis bears a very close resemblance to linear stability analysis around an equilibrium of time-discrete dynamical systems.

### Invasion and selection strength

Mutant fixation probabilities are a selection metric of great interest for graphs. However, they are analytically accessible only in a few special cases, e.g. [2, 25], and a computational approach for general graphs is only feasible when these are small [26], leading to a situation where simulations are widely used. It would thus be interesting to extrapolate fixation chances from invasion analysis, where the latter is easily performed with the method exposed in the previous section. Establishing whether invasion is successful or not may help to predict whether a mutant is neutral or not, which may be not an easy task for directed graphs of any size with node-dependent fitness. More exactly, it is expected that a mutation has a fixation probability higher than $\frac{1}{N}$ when Δλ > 0, while it has a fixation probability smaller than $\frac{1}{N}$ when Δλ < 0.

**Fig 2:**
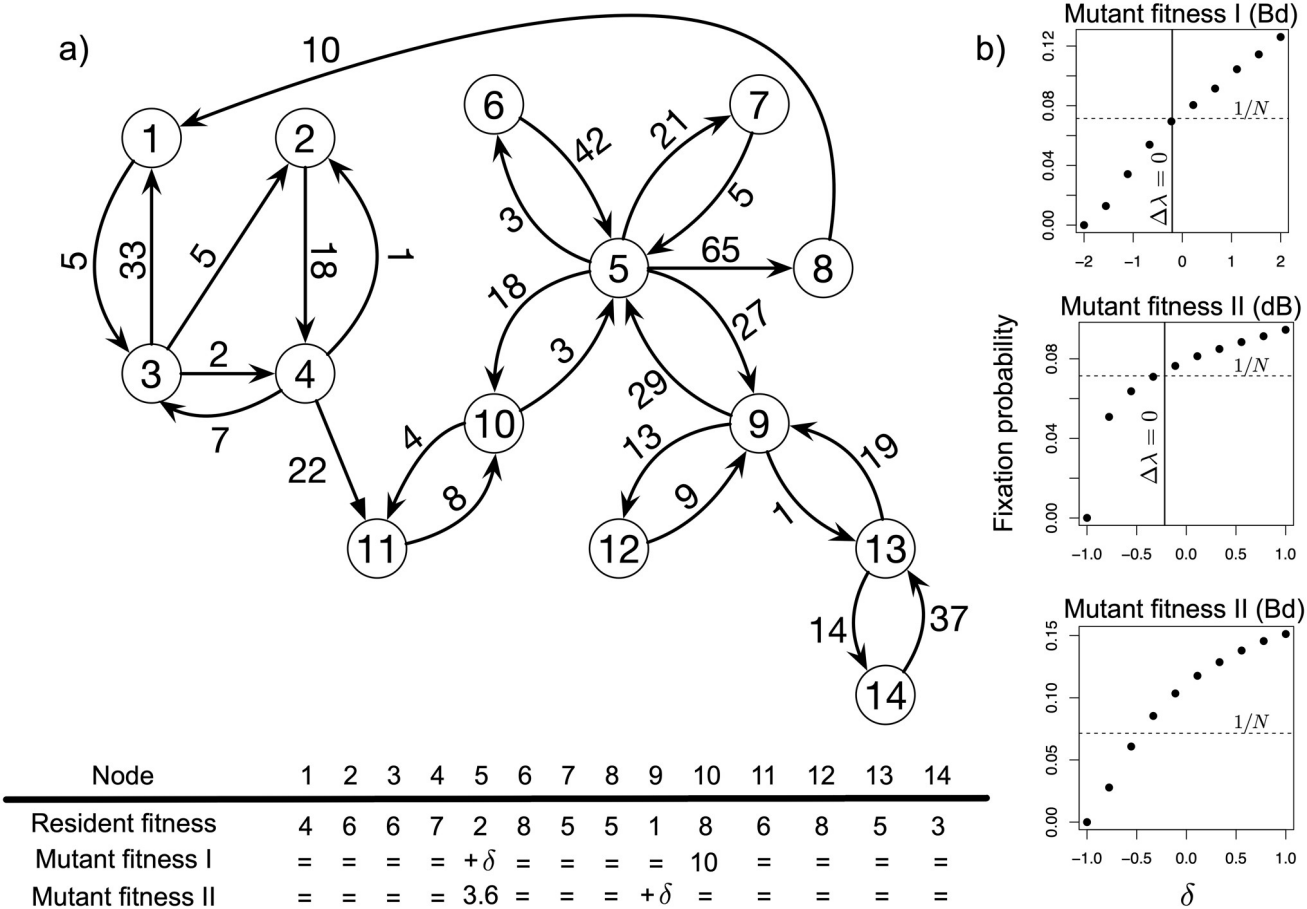
Invasion vs. fixation on a weighted directed graph. (a) The studied graph. Node dependent fitness for both types is found in the table just below. The mutant has greater fitness than the resident at a single node, while at another node the mutant has the same fitness as the resident plus some *δ*. Using the invasion criterion proposed in the main text (i.e. Δλ = 0) we predicted the value of *δ* required to make the mutant neutral. (b) the fixation probability for different *δ* values. Fixation probabilities were estimated from 20000 simulations of the process for each point for the first plots and 10^6^ simulations for the second and the third plot. The initial mutant is equally likely to appear in any one node. The horizontal dashed line gives the neutrality level (i.e. fixation probability equal to initial frequency). The vertical solid line identifies the neutral mutant according to our criterion. When invasion predicts fixation, fixation probabilities at the left of the intersection between the vertical (criterion) line and the horizontal (neutrality) line should be smaller than 1/*N* (i.e. deleterious mutation), while fixation probabilities at the right of this intersection should be greater than 1/*N* (i.e. beneficial mutation). In the third plot, invasion analysis erroneously classifies all mutants as successful invaders.

It is important to stress that, in general, the relationship between invasion and fixation is not trivial. Invasion analysis looks at expected mutant frequency change over a single time step just after mutant first appearance. The fixation probability integrates information over all possible paths the mutation can take to make its way from being present in a single individual to having a unity frequency. Clearly, there may be cases in which invasion analysis does not convey all relevant information needed to establish fixation [22] and any extrapolation from invasion analysis to fixation probability estimation is of heuristic nature. Fig 2 shows an application of our invasion criterion to a weighted directed graph with node dependent fitness and its relationship with fixation probabilities.

In this application, the graph structure and the fitness vectors were chosen arbitrarily to ensure that intuition cannot be a guide. In some cases (first two plots in panel b of Fig 2), our invasion criterion compared against simulation results distinguishes deleterious mutants, i.e. those with a fixation probability smaller than initial frequency, from beneficial mutants, i.e. those with a fixation probability greater than initial frequency. These results also show that if a mutant has an increase of a given magnitude in fitness at one node (with respect to resident fitness at the same node), then a different magnitude of fitness decrease at some other node may be required to make the mutant effectively neutral. This second magnitude depends in some nontrivial way on the graph structure and on both resident and mutant fitness at other nodes.

But, as Fig 2 also shows, the invasion criterion is not infallible: sometimes it fails to distinguish deleterious mutations from beneficial ones. In the third plot of panel b, all mutations should invade, yet some are clearly deleterious. The problem has to do with selection strength. The neutral matrix **A** and the invasion matrices $\tilde{A}$ constructed for each mutant in this plot are entrywise quite close. The maximum absolute difference is ≈ 0.01, which should still be within the scope of (4). However, the mutant fitness vector (third vector in the table in Fig 2) shows that at node 5 the mutant has a fixed 80% increase in fitness with respect to the resident. Then, mutant fitness at node 9 goes from almost zero to twice as much as resident fitness at that node. When mutant fitness at node 9 is almost zero, the corresponding fixation probability must also go to zero because node 9 is a bottleneck. Having vanishing fitness at this node makes one part of the graph completely inaccessible for a mutant starting from the other part. Clearly, by increasing mutant fitness at node 9, the probability of fixation should increase correspondingly, as observed in the simulations. When mutant fitness matches resident fitness, such probability is for sure higher than neutral. Yet invasion analysis is blind to such considerations. According to it, the sharp fitness increase in node 5 dominates and is sufficient to lead to successful invasion, i.e. Δλ > 0, regardless of mutant fitness at node 9. This reveals that marked differences in node specific fitnesses between resident and mutant may reflect into small Δ*a*_*i*,*j*_, which would suggest the applicability of invasion analysis. Yet marked fitness differences can impact fixation probabilities in a way that is not always captured by invasion analysis. The problem should not necessarily manifest, as the second plot of panel b in Fig 2 shows. Here the same fitness vectors as in the third plot are used under a different update rule and the invasion analysis yields reasonable results. However, it seems safe to use this form of analysis by controlling the strength of selection at the level of fitness, i.e. resident and mutant should have similar fitness vectors, rather than at the level of Δ*a*_*i*,*j*_.

### Node independent fitness

An exact correspondence between invasion and fixation holds when fitness is node independent. Let us normalize resident fitness to 1 so that mutant fitness can be expressed as 1+ *s*. Then the mutant successfully (unsuccessfully) invades and fixates with higher (smaller) than neutral probability when *s* > (<)0. Therefore, one does not need the analysis proposed above to predict invasion. However, such analysis still delivers a relevant piece of information that may be used: the very initial expected growth rate of the mutant inside the resident population. This suggests the use of the following approximation

$$\begin{array}{c} \phi_{j}\approx \frac{1-\text{exp}(-2v_{j}\Delta \lambda /\sigma^{2})}{1-\text{exp}(-2N\Delta \lambda /\sigma^{2})} \end{array}$$ (7)

proposed in [18] for demographically structured populations under weak selection to obtain an educated guess of fixation probabilities on graphs. Here *ϕ*_*j*_ is the fixation probability of a single mutant with fitness advantage Δλ > 0 that is initially found in stage *j* within an otherwise resident population. The initial reproductive value *v*_*j*_ of the mutant is computed in the neutral population and so is the parameter *σ*^2^, which is called the demographic variance of the population [18]. An approximation that is structurally analogous to (7) is derived for certain undirected graphs in [27], where quantities equivalent to reproductive values are also computed and used as weights for the mutant initial frequency. In Eq (7), Δλ expresses the difference between the growth rate of a demographically stable resident population and the expected initial growth rate of the mutant subpopulation [18], where the former is assumed equal to unity. As the quantity Δλ defined in the section about invasion analysis reflects expected mutant growth over one time step at the beginning of invasion in a graph, it seems reasonable to equate it with the quantity denominated in the same way in (7). As for *σ*^2^, in the general demographic case it is obtained from the average matrix population model under stochastic demography [28, 29]. In our setting, *σ*^2^ is the variance in neutral RV-fitness and is computed (see S1 Text) as

$$\begin{array}{c} \begin{array}{cc} \sigma^{2} & =\frac{1}{N}\sum_{i}\sum_{j}a_{i,j}\left(1-a_{i,j}\right)v_{i}^{2}\\ & +\frac{2}{N}\sum_{i}\sum_{j\neq i}a_{j,i}\left(1-a_{i,i}\right)v_{i}v_{j}\\ & -\frac{1}{N}\sum_{m}\sum_{i\neq m}\sum_{j\neq i\neq m}a_{i,m}a_{j,m}v_{i}v_{j}. \end{array} \end{array}$$ (8)

In graphs we are interested in the fixation probability of a single mutant that may appear at any one node with equal likelihood. Thus, the reproductive value of the initial mutant is the average reproductive value, which is 1. The formula of interest is then

$$\begin{array}{c} \phi =\frac{1-\text{exp}(-2\Delta \lambda /\sigma^{2})}{1-\text{exp}(-2N\Delta \lambda /\sigma^{2})} \end{array}.$$ (9)

**Fig 3:**
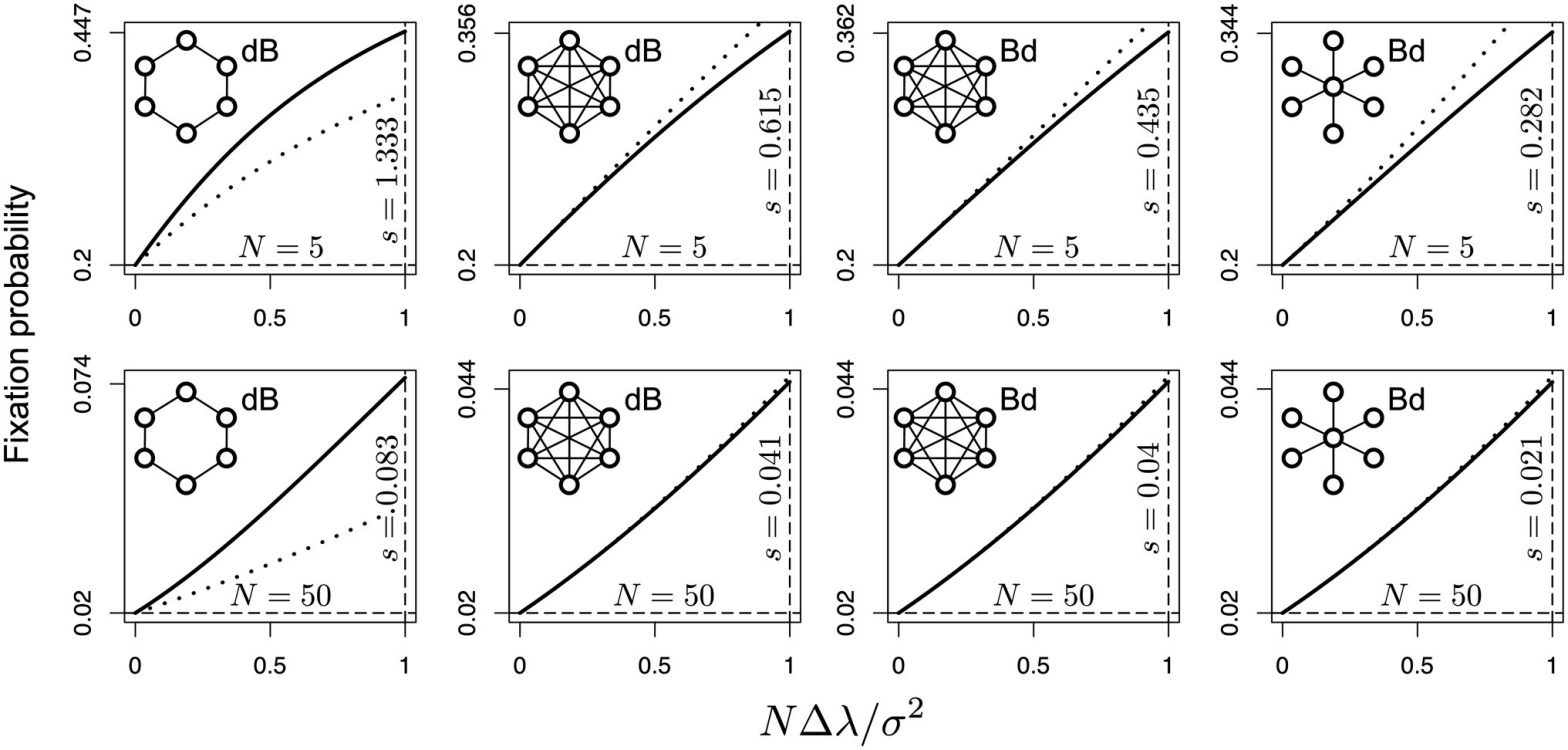
Comparison of approximate fixation probabilities (dotted line) of a beneficial mutant against exact results (solid line) in undirected graphs of different sizes. Fitness is node independent. Resident fitness is normalized to 1 and mutant fitness is 1 + *s*. The initial mutant is equally likely to appear in any node. The horizontal dashed line gives neutrality, i.e. 1/*N*. Along the vertical dashed line, the selective advantage *s* at *N*Δλ/*σ*^2^ = 1 is reported. Exact results for the complete graph under Bd and dB are given in [25]. Exact results for the star under Bd are given in [2]. Exact results for the cycle under dB are given in [30]. Approximations are computed from Eq (9) where the quantity Δλ is retrieved from the Perron root of $\tilde{A}$.

Fig 3 shows that this approximation compares well with exact results for some undirected graphs. To make results in this figure comparable across different structures, sizes and update processes, we looked at a regime of weak selection, i.e. as a rule of thumb, when *N*Δλ/*σ*^2^ ≪ 1. As explained in the next Section, we can think that *N*Δλ/*σ*^2^ ≈ *sN*_*e*_ where *N*_*e*_ is the population effective size.

As the approximation in (9) yields the probability of fixation from the very initial mutant growth, it implicitly assumes that fixation is not strongly influenced by the dynamics at intermediate or high mutant frequencies. Admittedly, this may not be justified for certain graph structures [8]. More in general, it should be kept in mind that the formula above has a heuristic components for two main reasons. First, it has not been directly derived for graphs starting from first principles, but it is an adaptation of a classic result in population genetics to demographically structured populations. Second, its reliance on the very initial mutant growth rate (from 1 to 2 mutants) fails to explicitly account for the possible long term dynamical effects of the clustering of mutants as the subpopulation of these gets larger. The results in Fig 3 make apparent some potential problems of the proposed approach in the case of the cycle graph with dB update.

### Drift in undirected graphs

In population genetics, the effective population size *N*_*e*_ quantifies genetic drift [31]. There are many definitions of *N*_*e*_ depending on the salient feature generating drift. Here we compute a “fixation” effective size for undirected graphs with node independent fitness. Following the approach in [4], *N*_*e*_ is here equated with the parameter scaling the difference *s* between resident individual fitness (1) and mutant individual fitness (1+ *s*, with *s* > 0) in a Kimura-like [32] approximation to the fixation probability of a slightly advantageous mutant, i.e.

$$\begin{array}{c} \phi =\frac{1-\text{exp}(-2sN_{e}/N)}{1-\text{exp}(-2sN_{e})}. \end{array}$$ (10)

Following a customary simplification of this formula [33], one first considers the case in which selection is weak and, then, large *N* is assumed (note that, in general, inverting this procedure leads to different results [34]) to get

$$\begin{array}{c} \phi \approx \frac{1}{N}+s\frac{N_{e}}{N}\frac{N-1}{N}\approx s\frac{N_{e}}{N}. \end{array}$$ (11)

In our framework, we first perform a first-order Taylor expansion around Δλ = 0 in (9), we expand Δλ to the first order around *s* = 0 and we then consider large *N*,

$$\begin{array}{c} \phi \approx \frac{1}{N}+s\frac{1}{\sigma^{2}}\frac{\partial \lambda }{\partial s}{|}_{s=0}\frac{N-1}{N}\approx s\frac{1}{\sigma^{2}}\frac{\partial \lambda }{\partial s}{|}_{s=0}. \end{array}$$ (12)

**Fig 4:**
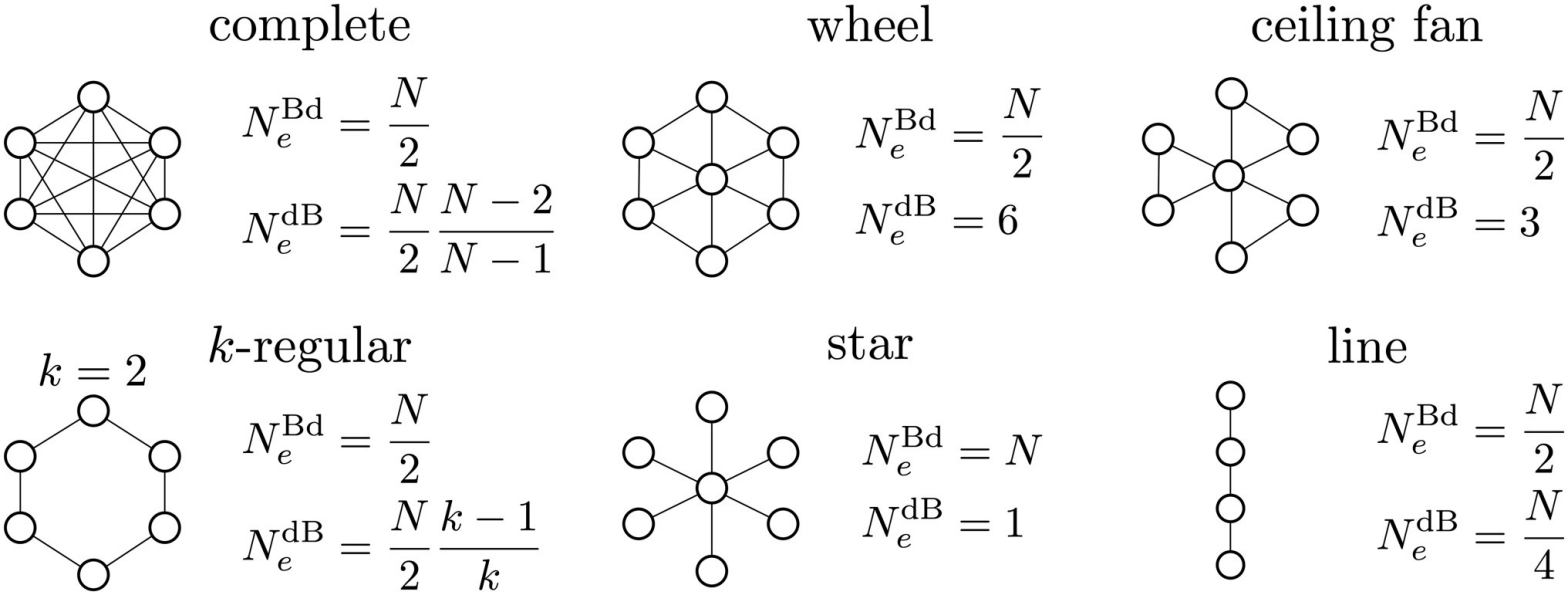
Effective size for large undirected graphs under both update processes.

Equating the expressions in (11) and (12) and solving for *N*_*e*_,

$$\begin{array}{c} N_{e}=\frac{N}{\sigma^{2}}\frac{\partial \lambda }{\partial s}{|}_{s=0} \end{array}$$ (13)

we get a measure of effective size in our context. Using this definition and considering large graphs, we find for the fixation probability

$$\begin{array}{c} \phi \approx s\frac{N_{e}}{N} \end{array}$$ (14)

which matches the classic approximation for large haploid populations [33]. Clearly, the same caveats as in the previous section apply. Fig 4 reports *N*_*e*_ under both update processes for several classes of graphs with large size (see S1 Text).

We note that the effective size of a complete graph under Bd is $\frac{N}{2}$ in agreement with previous results for other measures of effective size [4, 10, 35]. As expected, *k*-regular graphs of the same size have all the same fixation effective size under Bd. This is coherent with them having the same fixation probability for a given selective advantage [25]. Under dB, effective size may be a constant that depends on structure and not on size. This is consistent with dB inducing local competition. Here we give three immediate applications of our measure of effective size.

First, the star graph is a known selection amplifier under Bd [1]. Here we show that such amplification is explained by its having twice the effective size of a complete graph. In particular, our results in Fig 4 indicate that large stars have effective size equal to census size. We also find that, under dB, stars have an effective size of unity, which makes them very strong suppressors of selection consistently with results in [36]. Eq (13) can also be compared with an exact result for the fixation probability under Bd in stars found in [2]. Taking a first-order Taylor expansion at *s* = 0 of the exact result found in [2] and considering a large graph, we find *ϕ*_star_ ≈ *s*, which corresponds to our result.

**Fig 5:**
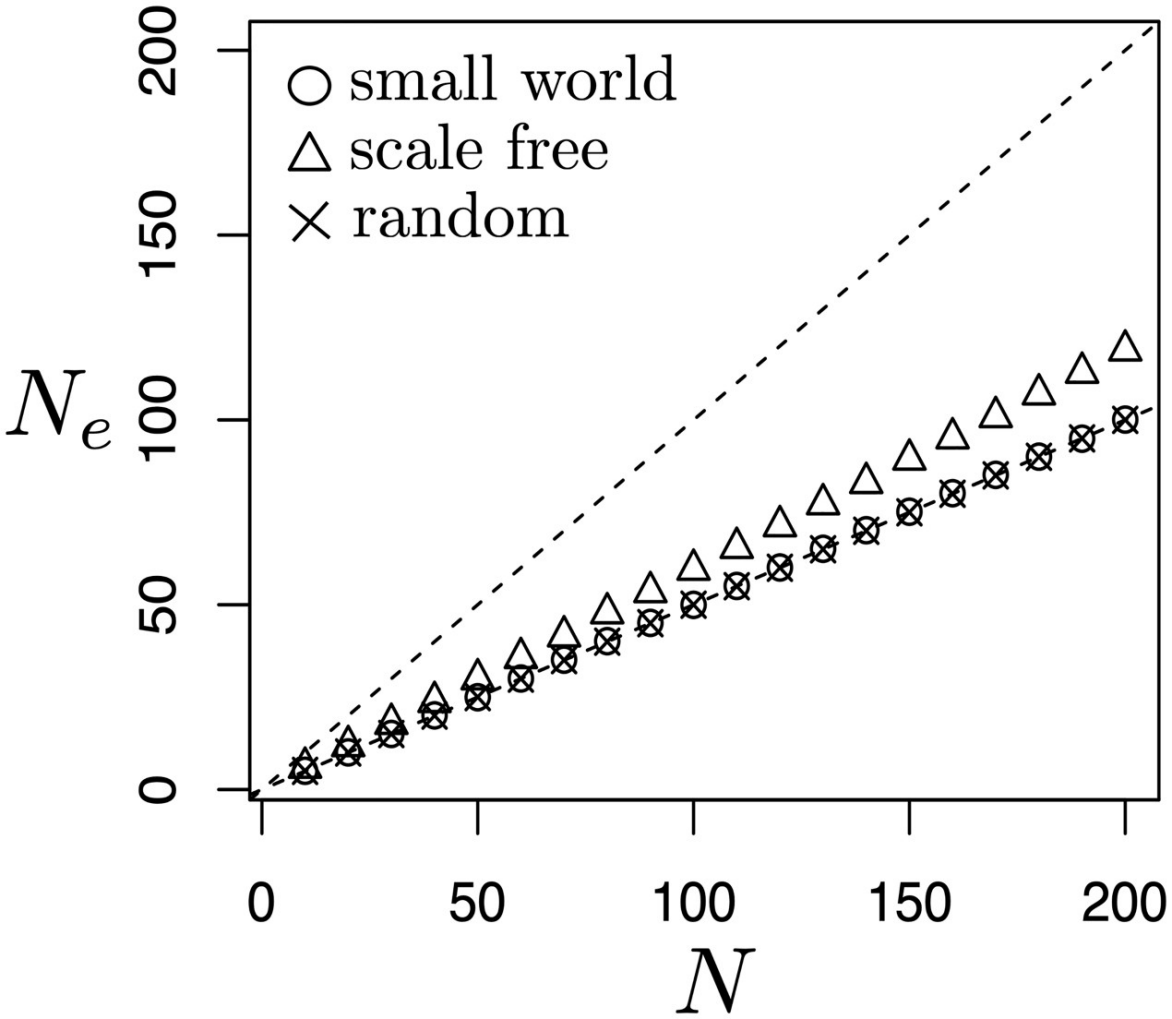
Effective size in networks under Birth-death update. The average effective size for three kinds of networks is computed in a random sample (with replacement) of 100 connected networks for each size and network kind. Dashed lines indicate *N*_*e*_ = *N* and $N_{e}=\frac{N}{2}$. For random networks, the probability of including an edge in the Erdős-Réyni algorithm was set to 0.75. For small-world networks, a 4-neighbors lattice was generated with a probability of 0.2 of link rewiring. Scale-free networks were generated by linear preferential attachment [39]. Random networks and small-world networks have an average effective size exactly aligned with that of a complete graph.

Second, Fig 4 shows that, under Bd and with large size, the effective size of the considered graphs tends to $\frac{N}{2}$, like in the complete graph, regardless of structure with the exception of the star. This is consistent with the conjecture that, under Bd, most large graphs have a fixation probability close to that of a complete graph [37]. Fig 5 reports the effective size of undirected graphs generated using the Erdős-Réyni algorithm (i.e. random graphs) and the Watts-Strogatz algorithm (i.e. small-world networks) [38].

We can see that the effective size is indeed $\frac{N}{2}$. Adopting the Barabási-Albert algorithm (i.e. scale-free networks, [39]), however, *N*_*e*_ is slightly greater than $\frac{N}{2}$. Hence, in the regime of weak selection, scale-free networks should confer greater fixation probability to a beneficial mutant if compared to a complete graph of the same size, as observed in simulations in [1].

Third, dB is a known suppressor of selection compared to Bd for complete undirected graphs [5]. Fig 4 shows that, between Bd and dB, the effective size of complete graphs differs by a factor $\frac{N-2}{N-1}$ explaining the reason behind selection suppression. To understand the precision of our approximation in large complete graphs, where the distinction between Bd and dB update becomes irrelevant, a first-order Taylor expansion of the exact result around *s* = 0 under either update process [5] leads to $\phi_{\text{complete}}\approx \frac{s}{2}$, which corresponds to our result using Eq (14). However, it should also be noted that the factor $\frac{k-1}{k}$ scaling $\frac{N}{2}$ under dB and not under Bd for *k*-regular graphs (see Fig 4) captures a known qualitative difference between the two update processes: dB tends to suppress selection compared to Bd [30]. Yet this factor quantitatively exaggerates the effect as Fig 3 shows in the case of the cycle.

## Discussion

Methods from evolutionary demography lend themselves to study evolution on graphs. Using them, we derived an invasion criterion for graphs with constant mutant fitness. Such criterion appears particularly useful in the presence of node dependent fitness and weighted graphs, when it may be unclear from a comparison of mutant and resident fitness vectors which will prevail. Moreover, the elaborated criterion can be adapted for both Birth-death and death-Birth updating. Intriguingly, for a given graph and for given resident and mutant fitness vectors, one can apply the criterion in both directions, i.e. checking for invasion of a single mutant in a resident population and for invasion of a single resident in a mutant population. If invasion is successful in both directions, it is possible to predict some form of coexistence. As dynamics are stochastic, however, the system will not persist at some interior equilibrium. This consideration connects directly with the fact that, sometimes, selection is said to favor the mutant over the resident when the fixation probability of a single mutant inside a resident population is greater than the fixation probability of a single resident inside a mutant population [23]. As we have shown, a general approximation to the average fixation probability of a slightly advantageous mutation on any graph and either update rule is also possible in our framework. Using this approximation, the analysis based on comparing fixation probabilities can in principle be performed with the proposed methods. A natural extension of the present work is the incorporation of game-theoretic interactions such as those between cooperators and defectors and fitness is no longer constant as assumed here. The usefulness of reproductive value considerations in this scenario is illustrated by results in [12, 40]. We can then envisage that expanding upon our approach, which is built around the key notion of reproductive value, may turn out helpful in the case of frequency dependent fitness.

Within the scope of the proposed approximation, which is limited in not accounting for evolutionary dynamics occurring at higher than vanishing mutant frequencies, we can also quantify drift on graphs via a measure of effective size. This measure recapitulates previously derived results [2, 4, 10, 30, 35]. Our effective size should, however, be seen distinct from, and complementary with the measures of (inbreeding and variance) effective size for undirected graphs under Bd elaborated in [10], which to our knowledge is the only other work so far addressing the problem of getting analytical expressions for the effective size of graphs. Although all measures provide the same result when applied to complete graphs ($N_{e}=\frac{N}{2}$), the measures in [10] are primarily meant to capture the average time conditional to fixation in graphs. Accordingly, they assign to an undirected cycle graph a much larger effective size than a complete graph under Bd. This reflects the fact that cycles have a larger fixation time than complete graphs [7, 8]. The effective size presented here instead is based on fixation probabilities. As cycles and complete graphs have the same fixation probability for a single mutant under Bd [1], they appear to have the same effective size on the basis of our *N*_*e*_.

The application of the proposed methods to undirected graphs with node independent fitness, which represent the most studied case, leads to particularly simple expressions that enhance our understanding of their evolutionary dynamics. More in general, our work makes explicit the connections between evolutionary graph theory and more classical population-genetic concepts. We hope this will help the future interplay of these two disciplines.

## Supporting information

S1 Text Update processes and derivation of key quantities. (PDF)
