# Supplementary material for: Invasion and effective size of graph-structured populations
Source: PLoS Comput Biol. 2018 Nov 12;14(11):e1006559. doi: 10.1371/journal.pcbi.1006559 (PMC6258371; doi:10.1371/journal.pcbi.1006559)
Supplement: S1 Text — (PDF) [file pcbi.1006559.s001.pdf]

S1 Text: Invasion and effective population size of graph-structured populations

Stefano Giaimo[1], Jordi Arranz[1], and Arne Traulsen[1]

[1]Department of Evolutionary Theory
Max Planck Institute for Evolutionary Biology
August-Thienemann-Straße 2, 24306 Plön, Germany
giaimo@evolbio.mpg.de

Update processes

Let $\mathbf{W} = [w_{i,j}]$ with $w_{i,j} \geq 0$ and $w_{i,i} = 0$ be the $N \times N$ matrix representing an evolutionary graph of N nodes, where $w_{i,j}$ is the weight on the arrow going from node j to node i. We observe the graph at discrete time steps $t = 0, 1, 2, \ldots$. As defined in the main text, $M(t) \subseteq U = \{1, 2, ..., N\}$ gives the current mutant configuration on the graph. Residents have fitness $\mathbf{z} = [z_1, z_2, ..., z_N]^T$ where $z_j > 0$ is the fitness of a resident at node j. Mutants have fitness $\mathbf{r} = [r_1, r_2, ..., r_N]^T$, where $r_j > 0$ is the fitness of a mutant at node j. Under Bd, the quantity $y_{i,j}(t)$ in Eq. (1) of the main text is

$$y_{i,j}(t, \mathrm{Bd}) = \begin{cases} \dfrac{z_j}{\sum\limits_{k \in M(t)} r_k + \sum\limits_{k \notin M(t)} z_k} \dfrac{w_{i,j}}{\sum\limits_k w_{k,j}} & \text{if } j \text{ is a resident at } t \\ \dfrac{r_j}{\sum\limits_{k \in M(t)} r_k + \sum\limits_{k \notin M(t)} z_k} \dfrac{w_{i,j}}{\sum\limits_k w_{k,j}} & \text{if } j \text{ is a mutant at } t \end{cases} \tag{1}$$

while under dB is

$$y_{i,j}(t, \mathrm{dB}) = \begin{cases} \dfrac{1}{N} \dfrac{w_{i,j} z_j}{\sum\limits_{k \in M(t)} w_{i,k} r_k + \sum\limits_{k \notin M(t)} w_{i,k} z_k}, & \text{if } j \text{ is a resident at } t \\ \dfrac{1}{N} \dfrac{w_{i,j} r_j}{\sum\limits_{k \in M(t)} w_{i,k} r_k + \sum\limits_{k \notin M(t)} w_{i,k} z_k}, & \text{if } j \text{ is a mutant at } t \end{cases} \tag{2}$$

Demographic variance

The demographic variance σ^2 is the expected variance in individual contribution to reproductive value in the next time step [1]. Let $\hat{v}_j = \sum_i v_i b_{i,j}$ be the contribution to reproductive value in the next time step of an individual in node j. Here $b_{i,j}$ is a random variable for the number of individuals contributed to node i by an individual in node j. Therefore, $\hat{v}_j$ is also a random variable with

	Birth-death (Bd)	death-Birth (dB)
complete	$\frac{2}{N}$	$\frac{2}{N}$
k-regular	$\frac{2}{N}$	$\frac{2}{N}$
star	$\frac{N-1}{N}\frac{N(2N+1)-2}{[(N-1)^2+1]^2} \sim \frac{2}{N^2}$	$\frac{2N^2+N-2}{4N(N-1)} \to \frac{1}{2}$
wheel	$\frac{4N^3-6N^2+18N-16}{3((N-1)^2+3)^2} \sim \frac{4}{3N}$	$\frac{N+8}{8(N-1)} \to \frac{1}{8}$
ceiling fan	$\frac{(N-1)(N^2+3)}{(2+(N-1)^2)^2} \sim \frac{1}{N}$	$\frac{2N^2+4N-6}{9(N-1)^2} \to \frac{2}{9}$
line	$\frac{2N+6}{(N+2)^2} \sim \frac{2}{N}$	$\frac{2N-3}{(N-1)^2} \sim \frac{2}{N}$

Table 1: Demographic variance σ^2 for the five undirected graphs shown in Fig. 4 in the main text and their asymptotic for $N \to \infty$.

expectation $\mathbb{E}(\hat{v}_j) = \lambda v_j$, stable population growth times reproductive value at j (see main text). The corresponding variance is [3]

$$\mathrm{Var}(\hat{v}_j) = \sum_i v_i^2 \mathrm{Var}(b_{i,j}) + 2\sum_{i<k} v_i^2 v_k^2 \mathrm{Cov}(b_{i,j}, b_{k,j}) \tag{3}$$

The average variance of $\hat{v}_j$ over all individuals at demographic stability when between-individual contributions are assumed independent is [3]

$$\sigma^2 = \sum_j u_j \mathrm{Var}(\hat{v}_j) = \sum_j u_j \left[\sum_i v_i^2 \mathrm{Var}(b_{i,j}) + 2\sum_{i<k} v_i^2 v_k^2 \mathrm{Cov}(b_{i,j}, b_{k,j}) \right]. \tag{4}$$

The quantity σ^2 for a neutral graph of N nodes is computed from the average projection matrix $\mathbf{A}$. In this case, $u_j = \frac{1}{N}$ for all j. All $\mathrm{Var}(b_{i,j})$ terms in (4) are $\mathrm{Var}(b_{i,j}) = a_{i,j}(1 - a_{i,j})$, because $a_{i,j}$ is the mean of $b_{i,j}$, which in graphs can only take value 0 or 1 under both update processes, and $a_{i,j}(1 - a_{i,j})$ is the corresponding variance. We now look at the covariance terms. Stasis/survival at node j and fertilities at the same node have positive covariance, as reproduction is conditional on survival. Let $b_{j,j}$ and $b_{i,j}$ be indicators for stasis in node j and offspring placement in node $i \neq j$, respectively. Then, $\mathbb{E}(b_{j,j}) = a_{j,j}$, $\mathbb{E}(b_{i,j}) = a_{i,j}$ and $\mathbb{E}(b_{j,j}b_{i,j}) = a_{i,j}$. Using $\mathrm{Cov}(x, y) = \mathbb{E}(xy) - \mathbb{E}(x)\mathbb{E}(y)$, we obtain $\mathrm{Cov}(b_{j,j}, b_{i,j}) = a_{i,j}(1 - a_{j,j})$. As for off-diagonal entries of column j, fertilities in node j have negative covariance between them, because in a reproduction event the offspring can be placed in only one node. Let $b_{i,j}$ and $b_{k,j}$ be indicators for offspring placement in i and offspring placement in k (where $i \neq j$, $k \neq i$ and $k \neq j$), respectively. Then, $\mathbb{E}(b_{i,j}b_{k,j}) = 0$ and $\mathrm{Cov}(b_{i,j}, b_{k,j}) = -a_{i,j}a_{k,j}$. Substituting in Eq. (4) leads to the expression for the demographic variance of a graph in the main text. In Table 1, we report formulas for σ^2 for several undirected graphs.

Undirected graphs with node independent fitness

In undirected graphs with node independent fitness, residents have fitness 1, while beneficial mutants have fitness $1 + s$ with $s > 0$. Then $\mathbf{W}$ is the graph

adjacency matrix and $w_{i,j}$ is an indicator variable that takes value 0 or 1 depending on the absence or presence, respectively, of a link between i and j. Let k_j be the degree (i.e. number of links) of node j. Then, for neutral populations ($s = 0$) under Bd the probability that j is selected to reproduce is $\frac{1}{N}$ for all j and the probability that j will place the offspring in node $i \neq j$ is $\frac{1}{k_j}$. Hence,

$$a_{i,j} = \begin{cases} \dfrac{w_{i,j}}{Nk_j} & i \neq j \text{ and process is Bd} \\ 1 - \dfrac{1}{N}\sum_m \dfrac{w_{i,m}}{k_m} & i = j \text{ and process is Bd} \end{cases} \tag{5}$$

Under dB, i dies with probability $\frac{1}{N}$ and each neighbor has a probability $\frac{1}{k_i}$ of replacing i with an offspring. Hence,

$$a_{i,j} = \begin{cases} \dfrac{w_{i,j}}{Nk_i} & i \neq j \text{ and process is dB} \\ 1 - \dfrac{1}{N} & i = j \text{ and process is dB} \end{cases} \tag{6}$$

The reproductive value of i is proportional to k_i^{-1} under Bd and to k_i under dB [2]. In our scaling,

$$v_i = \begin{cases} \dfrac{N}{k_i \sum_j \frac{1}{k_j}} & \text{process is Bd} \\ \dfrac{Nk_i}{\sum_j k_j} & \text{process is dB} \end{cases} \tag{7}$$

Table 2 reports reproductive values for graphs in Fig. 4 of the main text.

To get $\Delta\lambda$, we slightly perturb the matrix $\mathbf{A}$ by s. Differentiating and evaluating at $s = 0$ leads to linear mutant deviations,

$$\Delta a_{i,j} = \begin{cases} s\dfrac{(N-1)w_{i,j}}{N^2 k_j} & i \neq j \text{ and process is Bd} \\ s\dfrac{1}{N^2}\sum_m \dfrac{w_{i,m}}{k_m} & i = j \text{ and process is Bd} \end{cases} \tag{8}$$

and

$$\Delta a_{i,j} = \begin{cases} s\dfrac{(k_i - 1)w_{i,j}}{k_i^2 N} & i \neq j \text{ and process is dB} \\ 0 & i = j \text{ and process is dB} \end{cases} \tag{9}$$

Using Eq. (4) from the main text,

$$\Delta\lambda = \frac{1}{N}\sum_{i,j} v_i \Delta a_{i,j} \tag{10}$$

Table 3 reports computations of $\Delta\lambda$ using this formula for graphs in Fig. 4.

Alternatively, the matrices $\tilde{\mathbf{A}}$ can be formed without expressing mutant values as linear deviations from resident values. The quantity $\Delta\lambda$ can be retrieved from the Perron root of these matrices. Under Bd, a single mutant in an otherwise resident population has a probability $\frac{1+s}{N+s}$ of being selected for reproduction, while a resident individual has corresponding probability $\frac{1}{N+s}$.

	reproductive value (Bd)	reproductive value (dB)
complete	$v_j = 1$	$v_j = 1$
k-regular	$v_j = 1$	$v_j = 1$
star	$v_{\text{center}} = \frac{N}{(N-1)^2+1}$<br>$v_{\text{periphery}} = \frac{N(N-1)}{(N-1)^2+1}$	$v_{\text{center}} = \frac{N}{2}$<br>$v_{\text{periphery}} = \frac{N}{2(N-1)}$
wheel	$v_{\text{center}} = \frac{3N}{(N-1)^2+3}$<br>$v_{\text{periphery}} = \frac{N(N-1)}{(N-1)^2+3}$	$v_{\text{center}} = \frac{N}{4}$<br>$v_{\text{periphery}} = \frac{3N}{4(N-1)}$
ceiling fan	$v_{\text{center}} = \frac{2N}{(N-1)^2+2}$<br>$v_{\text{periphery}} = \frac{N(N-1)}{(N-1)^2+2}$	$v_{\text{center}} = \frac{N}{3}$<br>$v_{\text{periphery}} = \frac{2N}{3(N-1)}$
line	$v_{\text{extremity}} = \frac{2N}{N+2}$<br>$v_{\text{interior}} = \frac{N}{N+2}$	$v_{\text{extremity}} = \frac{N}{2(N-1)}$<br>$v_{\text{interior}} = \frac{N}{N-1}$

Table 2: Reproductive values for the undirected graphs shown in Fig. 4.

	$\partial\lambda/\partial s\|_{s=0}$ (Bd)	$\partial\lambda/\partial s\|_{s=0}$ (dB)
complete	$\frac{1}{N}$	$\frac{1}{N}\frac{N-2}{N-1} \sim \frac{1}{N}$
k-regular	$\frac{1}{N}$	$\frac{1}{N}\frac{k-1}{k}$
star	$\frac{2N(N-1)}{N^2[(N-1)^2+1]} \sim \frac{2}{N^2}$	$\frac{N-2}{2N(N-1)} \sim \frac{1}{2N}$
wheel	$\frac{2(N-1)[(N-1)^2+4N-1]}{3N^2[(N-1)^2+3]} \sim \frac{2}{3N}$	$\frac{3N-4}{4N(N-1)} \sim \frac{3}{4N}$
ceiling fan	$\frac{(N-1)[(N-1)^2+5N-1]}{2N^2[(N-1)^2+2]} \sim \frac{1}{2N}$	$\frac{2N-3}{3N(N-1)} \sim \frac{2}{3N}$
line	$\frac{N+1}{N(N+2)} \sim \frac{1}{N}$	$\frac{N-2}{2N(N-1)} \sim \frac{1}{2N}$

Table 3: Sensitivity of λ to a small change in node-independent fitness for graphs in Fig. 4 and their asymptotic behavior for $N \to \infty$.

	Selective RV-advantage $\Delta\lambda$ (Bd)
complete	$\frac{s}{N+s}$
k-regular graph	$\frac{s}{N+s}$
star	$\frac{1}{N+s}\left[1-\frac{N^2}{2(N-1)}+\sqrt{(1+s)^2+\left(\frac{N(N-2)}{2(N-1)}\right)^2}\right]$
wheel	$\frac{1}{6(N+s)}\left[2(1+s)-\frac{N^2-2}{N-1}+1+s\sqrt{\left(4+\frac{N(N-4)}{(1+s)(N-1)}\right)^2-\frac{4N(N-4)}{(1+s)(N-1)}}\right]$
ceiling fan	$\frac{1}{4(N+s)}\left[1+s-\frac{N^2-N+2}{N-1}+1+s\sqrt{\left(3+\frac{N(N-3)}{(1+s)(N-1)}\right)^2-\frac{4N(N-3)}{(1+s)(N-1)}}\right]$

Table 4: Selective RV-advantage under Bd computed from the Perron root of the matrix $\tilde{\mathbf{A}}$.

	Selective RV-advantage $\Delta\lambda$ (dB)
complete	$\frac{s(N-2)}{N(N-1+s)}$
k-regular graph	$\frac{s(k-1)}{N(k+s)}$
star	$\frac{1}{N}\left(\sqrt{\frac{(1+s)(N-1)}{N-1+s}}-1\right)$
wheel	$\frac{1}{N}\left[\frac{1+s}{3+s}\left(1+\sqrt{\frac{s+4(N-1)}{N-1+s}}\right)-1\right]$
ceiling fan	$\frac{1}{N(2+s)}\left[\frac{1+s}{2}\left(\sqrt{\frac{5N+(4N-3)(1+s)-6}{N-1+s}}-1\right)-1\right]$

Table 5: Selective RV-advantage under dB computed from the Perron root of the matrix $\tilde{\mathbf{A}}$.

Hence,

$$\tilde{a}_{i,j} = \begin{cases} \dfrac{(1+s)w_{i,j}}{(N+s)k_j} & i \neq j \text{ and process is Bd} \\ 1 - \dfrac{1}{N+s}\displaystyle\sum_{m\neq i}\dfrac{w_{i,m}}{k_m} & i = j \text{ and process is Bd} \end{cases} \tag{11}$$

Under dB, a single mutant in j within an otherwise resident population has a probability $\frac{1+s}{k_i+s}$ of being selected to reproduce given $i \neq j$ is selected to die. Hence,

$$\tilde{a}_{i,j} = \begin{cases} \dfrac{(1+s)w_{i,j}}{N(k_i+s)} & i \neq j \text{ and process is dB} \\ 1 - \dfrac{1}{N} & i = j \text{ and process is dB} \end{cases} \tag{12}$$

Note that the left eigenvector problem $\mathbf{c}\tilde{\mathbf{A}} = \lambda\mathbf{c}$ may sometimes be simplified to fewer than N equations leading to an explicit expression for $\tilde{\lambda}$ (the Perron root). This is done by noting that $\mathbf{c}$ is the invading mutant reproductive value vector. A regular graph appears identical from every node to a single invader and mutant reproductive value must be equal at all nodes. Then $\mathbf{c}$ has all components scaled to 1 and the eigenvector problem reduces to a single linear equation in λ. In star, ceiling fun and wheel graphs, we have only two classes of nodes and, similarly, we obtain a reduction from N to 2 equations (a quadratic problem in λ where $\tilde{\lambda}$ is the greatest root). This is done by noting that mutant reproductive value can only be different between the central node and any periphery node. Tables 4-5 report $\Delta\lambda$ computed with this approach for several graph structures. In the case of the line, the strategy is not as effective. Let us number nodes from 1 to N consecutively along the line. If N is even, then nodes j and $N-j+1$ (with $1 \leq j \leq \frac{N}{2}$) look identical to an invading mutant. Every node is in one of such pairs. Therefore, the possible reduction in the number of equations is from N to $\frac{N}{2}$ If N is odd, then nodes j and $N-j+1$ (with $1 \leq j \leq \frac{N-1}{2}$) look identical to an invading mutant. Every node is in one of such pairs except node $\frac{N-1}{2}+1$, which should be treated separately. Therefore, the reduction in the number of equations is from N to $\frac{N-1}{2}+1$.

References

[1] Steinar Engen, Russell Lande, Bernt-Erik Sæther, and F Stephen Dobson. Reproductive value and the stochastic demography of age-structured populations. *The American Naturalist*, 174(6):795–804, 2009.

[2] W. Maciejewski. Reproductive value on evolutionary graphs. *Journal of Theoretical Biology*, 340:285–293, 2014.

[3] Yngvild Vindenes, Aline Magdalena Lee, Steinar Engen, and Bernt-Erik Sæther. Fixation of slightly beneficial mutations: effects of life history. *Evolution*, 64:1063–1075, 2009.
